# Bacterial and fungal bloodstream infections in pediatric liver and kidney transplant recipients

**DOI:** 10.1186/s12879-021-06224-2

**Published:** 2021-06-08

**Authors:** Dina Leth Møller, Søren Schwartz Sørensen, Neval Ete Wareham, Omid Rezahosseini, Andreas Dehlbæk Knudsen, Jenny Dahl Knudsen, Allan Rasmussen, Susanne Dam Nielsen

**Affiliations:** 1 Viro-immunology Research Unit, Department of Infectious Diseases 8632, Rigshospitalet, University of Copenhagen, Blegdamsvej 9B, DK-2100 Copenhagen Ø, Denmark; 2 Department of Nephrology, Rigshospitalet, University of Copenhagen, Copenhagen, Denmark; 3 Department of Clinical Medicine, University of Copenhagen, Copenhagen, Denmark; 4 Centre of Excellence for Health, Immunity, and Infections, Department of Infectious Diseases, Rigshospitalet, University of Copenhagen, Copenhagen, Denmark; 5 Department of Cardiology, Rigshospitalet, University of Copenhagen, Copenhagen, Denmark; 6 Department of Clinical Microbiology, Rigshospitalet, University of Copenhagen, Copenhagen, Denmark; 7 Department of Surgical Gastroenterology and Transplantation, Rigshospitalet, University of Copenhagen, Copenhagen, Denmark

**Keywords:** Liver transplantation, Kidney transplantation, Bacteremia, Fungemia

## Abstract

**Background:**

Bacterial and fungal bloodstream infections (BSI) are common after pediatric liver and kidney transplantations and associated with morbidity and mortality. However, knowledge about incidence rates, pathogen composition, and resistance patterns is limited. We aimed to describe the pattern of bacterial and fungal BSI in a cohort of pediatric liver and kidney transplant recipients.

**Methods:**

A prospective study of 85 pediatric liver and kidney transplant recipients transplanted from 2010 to 2017 with a total of 390 person-years of follow-up. Clinical characteristics and BSI were retrieved from national registries assuring nationwide follow-up for at least 1 year. BSI incidence rates and pathogen composition were investigated and stratified by the time post-transplantation and type of transplanted organ.

**Results:**

A total of 29 BSI were observed within the first 5 years post-transplantation with 16 different pathogens. The overall incidence rate of first BSI was 1.91 per 100 recipients per month (95% CI, 1.1–3.1) in the first year post-transplantation. The most common pathogens were *Enterococcus faecium, Candida albicans*, *Escherichia coli*, and *Klebsiella pneumoniae*. The pathogen composition depended on the transplanted organ with a higher proportion of BSI with Enterobacterales in kidney transplant recipients than in liver transplant recipients (67% vs. 20%, *p* = 0.03), while multiple pathogens were detected in the liver transplant recipients.

**Conclusions:**

BSI were common in pediatric liver and kidney transplant recipients and the pathogen composition differed between liver and kidney transplant recipients. Guidelines for empiric antibiotic therapy should consider the type of transplanted organ as well as the local resistance patterns.

**Supplementary Information:**

The online version contains supplementary material available at 10.1186/s12879-021-06224-2.

## Introduction

The outcome of liver and kidney transplantations in children has improved over the last decades with a 5-year survival of 85–96% [1, 2]. However, complications to immunosuppression including severe infections and malignancies still cause substantial morbidity and mortality in these recipients [1, 3, 4]. Infections are associated with allograft dysfunction [1] and are a common cause of death in children after liver and kidney transplantation [1, 4, 5].

Bacterial and fungal bloodstream infections (BSI) are serious and prevalent infections. Thus, BSI have been reported in up to 25% of pediatric liver transplant recipients within the first year post-transplantation [6]. Knowledge about the composition of the pathogens that cause serious infections including BSI is important for the choice of empiric antibiotic therapy and may influence treatment outcome. In adult liver and kidney transplant recipients, the incidence rate (IR) of infections and the pathogen composition (bacteria, fungal, and virus) change during the first year post-transplantation [7]. Little is known about the pattern of infections over time in pediatric liver and kidney transplant recipients. Previous studies on the pathogens causing BSI in pediatric liver and kidney recipients have primarily focused on either the early post-transplantation period (< 6 months post-transplantation) [8–10] or the late post-transplantation period (> 6 months post-transplantation) [11] and studies with a complete follow-up are warranted.

This study was conducted to describe the pattern of bacterial and fungal BSI in pediatric liver and kidney transplant recipients and to provide information for recommendations regarding the use of empiric antibiotic therapy. We prospectively examined all bacterial and fungal BSI in pediatric liver and kidney transplant recipients at a tertiary pediatric transplantation center during the period 2010–2017 to evaluate the BSI incidence rates, BSI pathogen composition, and mortality after BSI during the first year post-transplantation.

## Materials and methods

### Study design and participants

In this prospective study, we included pediatric recipients (< 18 years of age) going through the first liver, kidney, or combined liver and kidney transplantation at Rigshospitalet, University Hospital of Copenhagen, Denmark, from January 1st, 2010 to December 31st, 2017. Rigshospitalet is a large tertiary hospital and specialist center of knowledge in transplantation and the only center in Denmark for pediatric liver transplantations. Pediatric liver and kidney transplant recipients were included from the Management of Posttransplant Infections in Collaborating Hospitals (MATCH) cohort [12].

Clinical characteristics including transplantation-specific data and microbiology were retrieved from the Centre of Excellence for Personalized Medicine of Infectious Complications in Immune Deficiency (PERSIMUNE) data repository [13]. Data were generated prospectively as part of the routine care and were merged in the PERSIMUNE data repository. The PERSIMUNE data repository contains data from several national registries and clinical databases including the Danish Microbiology Database (MiBa) that contains data on all microbiological samples from all Departments of Clinical Microbiology in Denmark with samples from both general practice and hospitals with complete coverage since 2010 [14]. From MiBa we received information about all blood cultures, including antibiotic resistance profiles, on all recipients from the day of transplantation to the end of follow-up.

The study was conducted in accordance with the declaration of Helsinki. The retrieval of the data was approved by the National Committee on Health Research Ethics (H-170024315) and the Data Protection Agency (04433, RH-2016-47).

### Immunosuppression and antimicrobial prophylaxis

The standard immunosuppressive regimen after liver and combined liver and kidney transplantation consisted of tacrolimus, mycophenolate mofetil (MMF), and prednisolone (Supplementary material content 1). The standard immunosuppressive regimen in kidney transplantation consisted of induction with basiliximab and tacrolimus, MMF, and prednisolone (Supplementary material content 1). All liver and combined liver and kidney transplant recipients received meropenem (15 mg/kg/3xday) in the first 5 days post-transplantation. Kidney transplant recipients received a single dose of cefuroxime (40 mg/kg) preoperatively. High-risk liver and combined liver and kidney (D^pos^/R^pos/neg^) and all kidney transplant recipients received 3 months of Cytomegalovirus prophylaxis with valganciclovir (dose depending on body surface area and creatinine). Low-risk liver and combined liver and kidney (D^neg^/R^pos/neg^) transplant recipients received 3 months of valaciclovir (dose depending on estimated glomerular filtration rate (eGFR)). Both liver, kidney, and combined liver and kidney transplant recipients received 6 months of trimethoprim-sulfamethoxazole (12.5/2.5 mg/kg/2xday 2 days a week) prophylaxis for *Pneumocystis jirovecii* infection. Neither the liver, kidney, nor combined liver and kidney transplant recipients received further standard prophylactic antifungal treatment. However, further antifungal treatment was used in liver and combined liver and kidney transplantation in certain situations including transplantation due to biliary atresia, acute surgery, and retransplantation among others.

### Bloodstream infection

The blood cultures were drawn on clinical indication. All blood cultures were analyzed using either BACTEC FX® (Becton, Dickinson and Company, Franklin Lakes, USA) or BACT/ALERT® (Biomérieux, Marcy l’Etoile, France) microbial detection systems. BSI was defined according to the Centers for Disease Control and Prevention (CDC) criteria [15] as an isolation of a bacterial or fungal pathogen from a blood culture that was not attributed to contamination unless the contaminant was isolated on two or more separate occasions within 2 days together with clinical symptoms of infections. *Corynebacterium spp*. (not *Corynebacterium diphtheria*), *Bacillus spp*. (not *Bacillus anthracis), Cutibacterium spp*., coagulase-negative staphylococci, non-hemolytic streptococci of the viridans group, *Aerococcus spp, and Micrococcus spp.* were regarded as contaminants [15]. Multiple episodes with the same pathogen were considered part of the same infection if they occurred within 14 days after the previous event [16].

Secondary BSI were defined using a modified version of the CDC criteria [16], where BSI were classified as secondary if they occurred within the secondary BSI attribution period (3 days before culture to 14 days after) of a culture with the same pathogen from another location than blood (for urine culture: Only cultures with ≥10^5^ CFU/ml).

The European Committee on Antimicrobial Susceptibility Testing (EUCAST) disk diffusion antimicrobial susceptibility testing method was used to investigate antibiotic resistance [17].

### Follow-up

All recipients were followed from the date of transplantation to the end of follow-up on December 31st 2018, retransplantation, or death, whichever came first. Inclusion stopped on December 31st 2017 to allow for at least 1 year of follow-up for all participants.

Number of BSI, BSI incidence rates, and pathogen composition were reported for the entire follow-up period and divided into four time periods post-transplantation; <1st month, 2nd-6th months, 7th–12th months, and 2nd-5th years post-transplantation. BSI occurring after 5 years of follow-up were not reported.

### Statistical analysis

In all analyses, liver and combined liver-kidney transplant recipients were merged as one group. Proportions were presented as percentages, and continuous data were presented as medians with range. The BSI incidence rates were calculated as the number of first BSI per recipient per person-time at risk in months to account for the different lengths of follow-up in both the liver and kidney transplant recipients and the different time periods post-transplantation. We calculated 95% confidence intervals (CI) using Byar’s approximation to the Poisson distribution. The 5-year cumulative incidence of first BSI for the combined group was calculated using the Aalen-Johansen estimator. Differences in pathogen composition of the BSI stratified by the transplanted organ were calculated using a Fisher’s exact test. The probability of survival after a BSI in the first year post-transplantation was calculated using a Kaplan-Meier estimator. All analyses were conducted in the statistical software R version 3.6.1 [18].

## Results

### Characteristics of the pediatric liver and kidney transplant recipients and the blood cultures

We included 85 pediatric liver, kidney, or combined liver and kidney transplant recipients into our cohort (Table 1) corresponding to all pediatric liver and combined liver and kidney transplantations and 46% of pediatric kidney transplantations performed in Denmark during the inclusion period. Liver and combined liver and kidney transplantations were more frequent (64%) than kidney transplantation (36%). The median age at the time of transplantation was 10.5 years (range 0.6–17.9) and there were slightly fewer males (49%) than females. Eight recipients (9%) had less than 1 year of follow-up due to early mortality or retransplantation. The median follow-up was 4.6 years (range: 0.01–8.9 years) with a total of 390 person-years of follow-up in the cohort.

**Table 1:** Patients characteristics

	All liver and kidney transplant recipients *n* = 85	Liver and combined liver-kidney transplant recipients *n* = 54 (64%)	Kidney transplant recipients *n* = 31 (36%)
		51 (94%) deceased-donor liver transplantations. 3 (6%) deceased-donor combined liver and kidney transplantations. 38 (75%) were split liver transplantations.	18 (58%) deceased-donor kidney transplantations. 13 (42%) living-donor kidney transplantations.
Age at transplantation, median (range)	10.5 (0.6–17.9)	10 (0.6–17.9)	11.8 (1.7–17.9)
Sex, male (%)	42 (49%)	25 (46%)	17 (55%)
Diseases leading to transplantation, Numbers of recipients (% of the recipients in the transplantation group)		Cholestatic disease including biliary atresia: 17 (31%)	Obstructive uropathy: 7 (22%)
		Metabolic disease: 9 (17%)	Hypo/dysplasia of the kidneys: 4 (13%)
		Cirrhosis including alfa-1-antitrypsin deficiency and cystic fibrosis: 8 (15%)	Congenital nephrosis: 3 (10%)
		Cancer: 7 (13%)	Glomerulonephritis and vasculitis: 3 (10%)
		Acute liver failure: 5 (9%)	Other: 5 (16%)
		Autoimmune hepatitis: 5 (9%)	Unknown: 9 (29%)
		Other: 3 (6%)	

A total of 883 blood cultures were drawn on 72 of 85 (85%) recipients during the first 5 years post-transplantation (Table 2). Of these, 59 blood cultures (7%) were positive, 805 blood cultures (91%) were negative, and 19 blood cultures (2%) were contaminated. The majority of blood cultures were drawn within the first year post-transplantation (64%).

**Table 2:** Blood cultures and bloodstream infections

	All liver and kidney transplant recipients *N* = 85	Liver and combined liver-kidney transplant recipients *N* = 54	Kidney transplant recipients *N* = 31
Number of analyzed blood culture^a^ (n of individual recipients (% of the cohort))	883 72 (85%)	735 47 (87%)	148 25 (81%)
Results of blood cultures			
- Positive	59 (7%)	48 (7%)	11 (7%)
- Contamination	19 (2%)	16 (2%)	3 (2%)
- Negative	805 (91%)	671 (91%)	134 (91%)
Bloodstream infections^b^ (n of individual recipients (% of the cohort))	29 19 (22%)	20 12 (22%)	9 7 (23%)
Secondary BSI			
- Primary (no previous cultivation (% of all BSI))	20 (69%)	16 (80%)	4 (44%)
- Secondary to cultivation in (% of all BSI)	9 (31%)	4 (20%)	5 (56%)
- Urine	5	–	5
- Drains of the abdomen	3	3	–
- Sputum	1	1	–
Incidence rate of first BSI per 100 recipients per month in the first year post-transplantation	1.91 (95% CI: 1.12–3.08)	2.1 (95% CI: 1.08–3.74)	1.61 (95% CI: 0.61–3.54)
Incidence rate of first BSI for all liver- and kidney transplant recipients	Per 100 recipients per month		
- 1st month	6.47 (95% CI: 2.45–14.17)		
- 2nd-6th month	2.55 (95% CI: 1.26–4.66)		
- 7th–12th month	0.45 (95% CI: 0.09–1.45)		
- 1st year	1.91 (95% CI: 1.12–3.08)		

^a^One Pediatric blood-culture flask

^b^After CDC criteria for BSI and 14 days repeat infection timeframe

The 59 positive blood cultures resulted in 29 BSI in 19 (22%) recipients (Table 2). None of the contaminated blood cultures comply with CDC criteria for BSI. Eleven (13%) recipients had 1 BSI, 6 (7%) recipients had 2 BSI, 2 (2%) recipients had 3 BSI during follow-up. Fifteen out of the 19 recipients with a BSI (79%), developed their first BSI within the first year post-transplantation. Five out of 29 (17%) BSI and 20 out of 29 (69%) BSI were observed in the first month and the first year post-transplantation, respectively.

### BSI incidence rate

The overall incidence rate (IR) of first BSI in the first year post-transplantation in the entire cohort was 1.91 first BSI per 100 recipients per month (95% CI, 1.1–3.1) corresponding to 22.5 first BSI per 100 recipients per year (95% CI, 13.2–36.2). The IR of first BSI was highest the first month post-transplantation (6.47 first BSI per 100 recipients per month (95% CI, 2.5–14.2)) and decreased throughout the year with a lower IR at 2nd-6th month (2.55 first BSI per 100 recipients per month (95% CI, 1.3–4.7)) and 7th–12th month post-transplantation 0.45 first BSI per 100 recipients per month (95% CI, 0.1–1.5)).

We observed both fungal (4 out of 20) and bacterial BSI (16 out of 20) in the liver transplant recipients whereas kidney transplant recipients only had bacterial BSI. The IR of first bacterial and first fungal BSI in the liver and combined liver and kidney transplant recipients was 1.67 (95% CI: 0.8-3.2) and 0.38 (95% CI: 0.1-1.2) per 100 recipients per month, respectively, in the first year post-transplantation. The IR of first bacterial in the kidney recipients was 1.61 (95% CI: 0.6-3.5) per 100 recipients per month in the first year post-transplantation. The IR of first BSI in the first year post-transplantation (combined bacterial and fungal BSI for the liver transplant recipients and bacterial only BSI for the kidney transplant recipients) is shown in Table 2.

**Fig. 1:**
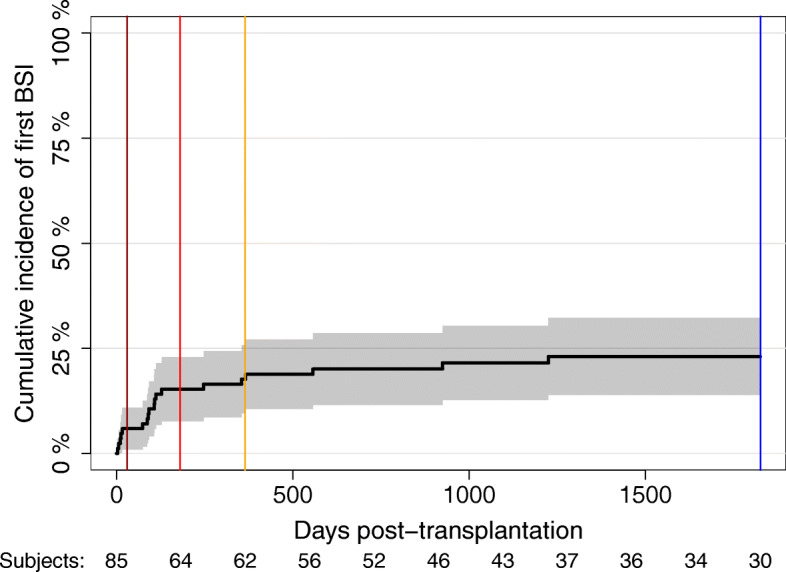
Cumulative incidence of first bloodstream infections (BSI) in the first 5 years post-transplantation in pediatric liver and kidney transplant recipients. Horizontal lines: Dark red is the first month, red is 6 months, yellow is the first year, and blue is the first 5 years post-transplantation

The cumulative incidence of first BSI in the first 5 years post-transplantation for the entire group was 23.06% (95% CI: 13.9-32.2) (Fig. 1).

### Pathogen composition in the BSI

**Fig. 2:**
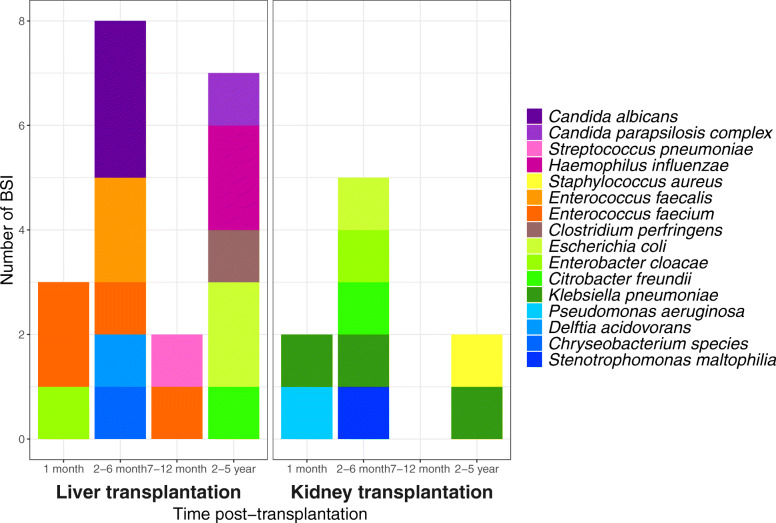
Pathogen composition from bloodstream infections (BSI) in pediatric liver and kidney transplant recipients in the first 5 years post-transplantation

In the 29 BSI observed in the first 5 years post-transplantation, 16 different pathogens were isolated (Fig. 2). The most common pathogens were *Enterococcus faecium* found in 4 out of 29 BSI (14%), whereas *Escherichia coli (E. coli)*, *Klebsiella pneumoniae (K. pneumoniae),* and *Candida albicans* each were found in 3 out of 29 BSI (10%). Nine BSI (31%) were classified as secondary BSI (Table 2). In the kidney transplant recipients, all secondary BSI were after a positive urine culture, whereas the liver transplant recipients had secondary BSI after positive cultures in drains of the abdomen (3/4) or sputum (1/4). The pathogen composition differed between liver and kidney transplant recipients (Fig. 2). There were significantly more BSI with Enterobacterales in kidney transplant recipients than in liver transplant recipients (6/9 (67%) vs. 4/20 (20%), *p* = 0.03). Furthermore, *Enterococci spp.* and *Candida spp*. were only observed in liver transplant recipients constituting 6 out of 20 (30%) and 4 out of 20 (20%) of BSI in this group.

### Antibiotic resistance profiles

Antibiotic resistance profiles were available for 27 out of 29 BSI (93%) (see Tables, Supplementary material content 2 and 3). Extended-spectrum-β-lactamase producing *E. coli* was observed in one BSI. There were no cultures with either methicillin-resistant *Staphylococcus aureus* or vancomycin- and/or linezolid resistant enterococci in the cohort. Among all bacterial BSI in the liver transplant recipients, 81% were susceptible to piperacillin/tazobactam and 81% to meropenem. Among all bacterial BSI in the kidney transplant recipients, 75% were susceptible to piperacillin/tazobactam and 88% to meropenem. All fungal BSI in the cohort were susceptible to fluconazole.

### Outcomes after transplantation

Five out of 54 (9%) liver transplant recipients and 1 out of 31 (3%) kidney transplant recipients died during the first year post-transplantation. Three (6%) liver transplant recipients were retransplanted during the first year post-transplantation. No recipients developed GvHD during the follow-up. One kidney transplant recipient (3%) was lost to follow-up since the recipient was not a Danish resident. One out of the 15 patients (7%), who develop a BSI within the first year post-transplantation, died in the first year post-transplantation. The probability of survival after a BSI in the first year post-transplantation was 93% (95% CI, 82–100%) with a median follow-up of 5.0 years.

## Discussion

This study was conducted to provide information about the pattern of BSI in pediatric liver and kidney transplant recipients. BSI were common with an incidence rate of 1.91 first BSI per 100 pediatric liver and kidney transplant recipients per month in the first year post-transplantation. The BSI pathogen composition differed between liver and kidney transplant recipients with bacteria from the Enterobacterales group being more common in kidney transplant recipients whereas *Candida spp*. and *Enterococci spp*. only were observed in liver transplant recipients.

No previous studies have reported the IR of BSI in pediatric liver and kidney transplant recipients during the entire first year post-transplantation. The overall IR of first BSI in the first year post-transplantation in our study is comparable with data from a nationwide Spanish study of 2935 adult solid organ transplant (SOT) recipients. In this report, the overall IR was 4.8 and 3.0 BSI per 10,000 days post-transplantation in liver and kidney transplant recipients, respectively [19] (corresponding to 1.5–0.9 BSI per 100 recipients per month). Furthermore, a recent study in adult SOT recipients showed that the IR of all infections in liver and kidney transplant recipients was highest during the early post-transplantation period and declined during the first year post-transplantation [7]. We found a similar trend in our cohort with a higher IR in the early post-transplantation period.

With regard to the pathogen composition, we found a higher frequency of BSI with Enterobacterales in kidney transplant recipients than in liver transplant recipients reflecting the high frequency of secondary BSI after urinary tract infection. Similar findings have been reported in adult liver and kidney transplant recipients where Gram-negative bacteria were the major cause of BSI in kidney transplant recipients [7, 19, 20]. The diversity in pathogen composition may also reflect the differences in diseases leading to transplantation in the two groups. We used CDC criteria for BSI which includes bacteria that are normally considered as contaminants including coagulase-negative staphylococci [15]. However, there were no BSI due to coagulase-negative staphylococci or other contaminants in our cohort. In contrast, previous studies that used CDC criteria reported as many as 30% of BSI being caused by coagulase-negative staphylococci in pediatric liver transplant recipients [6, 9]. Furthermore, we found only one case of *Staphylococcus aureus* which in previous retrospective studies has caused a high proportion of BSI [6, 21]. However, these studies were conducted in other geographical settings (Japan and the USA) where the antibiotic treatment strategies differ from the Danish approach which may, in part, explain the differences. Other explanations may include differences in surgical techniques, the use of intravenous catheters, and immunosuppression regimens between centers.

In our study, candidemia was rare and only found in liver transplant recipients with an IR of 0.38 fungal BSI per 100 recipients per month in the first year post-transplantation. This is in line with a study exclusively investigating invasive fungal disease in 584 pediatric SOT recipients that found no invasive fungal disease in the kidney group, while 27.5 invasive fungal diseases per 100,000 patients days (corresponding to 0.85 events per month per 100 recipients) was found in the liver transplant recipients [22]. However, this study only investigated invasive fungal disease in the first 180 days post-transplantation, and data for the later post-transplantation periods were not available. Since the majority of invasive candida infections have been reported to be from an endogenous source [23], the variation in IR between the liver and kidney transplant recipients may be caused by a higher frequency of candida colonization of the gut compared to the urinary tract.

Only one multidrug-resistant organism, an Extended-spectrum-β-lactamase (ESBL) producing *E. coli*, was observed in our cohort. This is in contrast to a study investigating severe sepsis in 173 pediatric liver transplant recipients reporting that 47.6% of the bacterial infections were with multidrug-resistant organisms [24]. However, this study only included a population of highly selected pediatric liver transplant recipients admitted to the pediatric intensive care unit. Furthermore, 24 out of 29 BSI (83%) were observed after the first month post-transplantation indicating that the majority of the BSI could be community-acquired, thus, lowering the risk of multidrug resistance. The incidence of community-onset ESBL producing *E. coli* and *K. pneumoniae* were found to be between 3-4% in 2010–2017 in a Danish study [25], supporting our finding of only one ESBL producing *E. coli* in the six *E.coli and K. pneumoniae* BSI.

Current reviews of BSI in solid organ transplant recipients including liver and kidney transplantation recommend empiric antibiotic treatment of suspected BSI depending on local epidemiology and previous microbiology data. The antibiotic regimens should cover Gram-negative bacteria and, in the case of intravascular catheters, also Gram-positive bacteria [26, 27]. In our center, the current recommendation for empiric antibiotic treatment of suspected BSI in pediatric liver and kidney transplant recipients is the use of carbapenem. Our findings support the use of empiric carbapenems in pediatric kidney transplant recipients since 88% of the BSI were susceptible to carbapenem. However, for recently transplanted (< 12 months) pediatric liver recipients, additional antibiotic coverage for *Enterococcus faecium* should be considered such as vancomycin or linezolid depending on local resistance patterns and previous history of vancomycin- and/or linezolid resistant enterococci.

Only one among the 15 recipients with a BSI in the first year post-transplantation died making statistical comparisons in mortality between patients with and without BSI difficult. We found that 8% of the BSI were due to *Streptococcus pneumoniae (S. pneumoniae)* and *Haemophilus influenzae*, which may be prevented by vaccination, highlighting the importance of vaccination prior to transplantation. A recent study of vaccine-preventable infections in 6980 pediatric SOT recipients reported infections with *S. pneumoniae* in 2% of the recipients and 17 times higher mortality after *S. pneumoniae* in SOT recipients compared to the general pediatric population [28].

Our study cohort included 68% of all pediatric liver and kidney transplant recipients transplanted in Denmark during the study period [29]. Nevertheless, groups were small making extrapolation difficult and highlighting the need for cross-national collaborations. Another limitation to the study is the lack of data on use of immunosuppressants and intravenous catheters as well as lack of information on vaccination status. The strengths of our study include the prospective design with long post-transplantation follow-up. The microbiology database MiBa is nationwide and includes all blood cultures on the recipients sampled in both in- and outpatient facilities as well as in general practice. Furthermore, the Danish Civil Registration System [30] ensures that the vital status is continuously followed for all recipients in the cohort, and we have a near-complete follow-up. Unfortunately, patients migrating and patients from the Faroe Islands and Greenland will be lost to follow-up. However, these patients only represent a small percentage of transplanted children in Denmark. The near-complete follow-up allows us to estimate the IR of BSI in our cohort with a small risk of underestimating the rate.

This study presents incidence rates and pathogen composition for BSI following pediatric liver and kidney transplantation. We found that BSI were common, with an incidence rate of 22.5 first BSI per 100 pediatric liver and kidney transplant recipients in the first year post-transplantation. The BSI pathogen composition differed between liver and kidney transplant recipients, and the empiric antibiotic treatment should depend on the type of transplanted organ and local epidemiology, including pathogen flora and resistance patterns.

## Supplementary Information

**Additional file 1: Supplementary material content 1.** Immunosuppressive regimen. **Supplementary material content 2.** Antibiotic resistance profiles for bacterial bloodstream infections. **Supplementary material content 3.** Antibiotic resistance profiles for fungal bloodstream infections

## Data Availability

The datasets generated and/or analyzed during the current study are not publicly available due to restrictions from the data protection law in Denmark but the data are available to be seen in person at our institution on reasonable request.

## Abbreviations

BSI: Bloodstream infections
CDC: Centers for Disease Control and Prevention
CI: Confidence intervals
*E. coli*: *Escherichia coli*
eGFR: Estimated glomerular filtration rate
EUCAST: The European Committee on Antimicrobial Susceptibility Testing
GvHD: Graft-vs-host disease
IR: Incidence rate
*K. pneumoniae*: *Klebsiella pneumoniae*
MATCH: Management of Posttransplant Infections in Collaborating Hospitals
MMF: Mycophenolate mofetil
MiBa: Danish Microbiology Database
PERSIMUNE: Centre of Excellence for Personalized Medicine of Infectious Complications in Immune Deficiency
SOT: Solid organ transplant
*Spp*.: Species pluralis
*S. pneumoniae*: *Streptococcus pneumoniae*
