# Supplementary material for: Bacterial and fungal bloodstream infections in pediatric liver and kidney transplant recipients
Source: BMC Infect Dis. 2021 Jun 8;21:541. doi: 10.1186/s12879-021-06224-2 (PMC8188646; doi:10.1186/s12879-021-06224-2)
Supplement: Supplementary file 1 — Additional file 1: Supplementary material content 1. Immunosuppressive regimen. Supplementary material content 2. Antibiotic resistance profiles for bacterial bloodstream infections. Supplementary material content 3. Antibiotic resistance profiles for fungal bloodstream infections [file 12879_2021_6224_MOESM1_ESM.docx]

**Supplementary material**

**Bacterial and fungal bloodstream infections in pediatric liver and kidney transplant recipients**

**Dina Leth Møller, MD ^a^; Søren Schwartz Sørensen, MD, DMsc. ^b,c^; Neval Ete Wareham, MD, PhD ^d^; Omid Rezahosseini, MD ^a^; Andreas Delbæk Knudsen, MD ^a,e^; Jenny Dahl Knudsen, MD, DMsc ^f^; Allan Rasmussen, MD ^g^; Susanne Dam Nielsen, MD, DMsc ^a,c *^.**

1. Viro-immunology Research Unit, Department of Infectious Diseases, Rigshospitalet, University of Copenhagen, Copenhagen, Denmark.

Dina.leth.moeller@regionh.dk (ORCID: 0000-0002-3909-0643)

Omid.rezahosseini@regionh.dk (ORCID: 0000-0003-2198-1904)

Andreas.dehlbaek.knudsen@regionh.dk (ORCID: 0000-0002-5349-7829)

sdn@dadlnet.dk (ORCID: 0000-0001-6391-7455)

1. Department of Nephrology, Rigshospitalet, University of Copenhagen, Copenhagen, Denmark.

Soeren.Schwartz.Soerensen@regionh.dk (ORCID: 0000-0001-5898-8048)

1. Department of Clinical Medicine, University of Copenhagen, Copenhagen, Denmark.
2. Centre of Excellence for Health, Immunity, and Infections, Department of Infectious Diseases, Rigshospitalet, University of Copenhagen, Copenhagen, Denmark.
   Neval.ete.wareham@regionh.dk (ORCID: 0000-0001-5746-9385)
3. Department of Cardiology, Rigshospitalet, University of Copenhagen, Copenhagen, Denmark.
4. Department of Clinical Microbiology, Rigshospitalet, University of Copenhagen, Copenhagen, Denmark.
   Inge.Jenny.Dahl.Knudsen@regionh.dk (ORCID: 0000-0002-8699-9956)
5. Department of Surgical Gastroenterology and Transplantation, Rigshospitalet, University of Copenhagen, Copenhagen, Denmark

Allan.Rasmussen@dadlnet.dk (ORCID: 0000-0002-9550-4767)

* Corresponding author at: Viro-immunology Research Unit, Department of Infectious Diseases 8632, Rigshospitalet, University of Copenhagen, Copenhagen, Denmark.

E-mail address: sdn@dadlnet.dk, Phone: (+45) 35 45 0859

Postal Address: Viro-immunology Research Unit, Department of Infectious Diseases 8632, Rigshospitalet, University of Copenhagen, Blegdamsvej 9B, DK-2100 Copenhagen Ø, Denmark.

**Supplementary material content 1**
*Immunosuppressive regimen*

The standard immunosuppressive regimen after liver and combined liver and kidney transplantation consisted of tacrolimus, mycophenolate mofetil (MMF), and prednisolone. The start dose of tacrolimus was 0.15 mg/kg/day administered in two doses. Target concentration were 8-10 ng/ml in the first month, 7-9 ng/ml in 2^nd^-3^rd^ month, 6-8 ng/ml in 3^rd^-6^th^ month, 5-7 ng/ml 6^th^-12^th^ month, and 4-6 ng/ml after the first-year post-transplantation. MMF was dosed as 1200 mg/m^2^/day administered in two doses in the first year and 600 mg/m^2^/day administered in two doses afterward. 1000 mg methylprednisolone was given intraoperatively. Prednisolone was tapered gradually from 1 mg/kg twice daily on the first-day post-transplantation to 0.2 mg/kg twice daily on day 6. For the remaining of the first month 0.3 mg/kg once daily was given. This was traped to 0.2 mg/kg once daily in months 2-3, 0.1 mg/kg in months 3-6, 0.05 mg/kg in months 6-12 after which the drug was discontinued.

The standard immunosuppressive regimen in kidney transplantation consisted of induction with basiliximab plus tacrolimus, MMF, and prednisolone. Baxilimab was administered before the transplantation and at day 4 posttransplantation. The start dose of tacrolimus was 0.1 mg/kg/day twice daily. Target concentrations were 5-10 ng/ml in the first 6 months post-transplantation and 4-7 ng/ml afterward. MMF was dosed as 450 mg/m^2^ twice daily. Prior to transplantation 10 mg/kg of prednisolone was given. Prednisolone was tapered gradually from 1,5 mg/kg/day in two doses on the first-day post-transplantation to 1 mg/kg in two doses on day 7. For the remaining of the first month 0.6 mg/kg/day was given. This was traped to 0.4 mg/kg/day in month 2, 0.3 mg/kg/day in month 3, 0.2 mg/kg/day in month 4-6, 0.15-0.2 mg/kg/day afterward.

**Supplementary material content 2.**
*Antibiotic resistance profiles for bacterial bloodstream infections*

| **Patient no.** | **Pathogen** | **Time from transplantation to BSI in days** | **Penicillin** | **Piperacillin/ Tazobactam** | **Cefuroxime** | **Ceftriaxone** | **Meropenem** | **Fluoroquinolones (Ciprofloxacin and Moxifloxacin)** | **Trimethoprim/ Sulfamethoxazole** | **Vancomycin** | **Linezolid** |
| --- | --- | --- | --- | --- | --- | --- | --- | --- | --- | --- | --- |
| 1 | Chryseobacterium species | 107 | - | S | - | - | S | S | - | - | - |
| 2 | Citrobacter freundii | 112 | - | S | - | I | S | S | - | - | - |
| 3 | Citrobacter freundii | 416 | - | S | R | I | S | S | S | - | - |
| 4 | Clostridium perfringens | 1225 | S | - | - | - | - | - | - | - | - |
| 1 | Delftia acidovorans | 107 | - | S | - | - | S | S | - | - | - |
| 5 | Enterobacter cloacae | 128 | R | I | I | I | S | S | R | - | - |
| 6 | Enterobacter cloacae | 11 | - | S | R | I | S | S | S | - | - |
| 7 | Enterococcus faecalis | 90 | I | S | R | R | S | R | - | S | S |
| 7 | Enterococcus faecalis | 125 | R | S | R | R | S | R | - | S | S |
| 8 | Enterococcus faecium | 108 | R | R | R | R | R | R | - | S | S |
| 9 | Enterococcus faecium | 6 | - | - | - | - | - | R | - | S | S |
| 3 | Enterococcus faecium | 355 | - | - | - | - | - | R | - | S | S |
| 10 | Enterococcus faecium | 3 | R | - | R | R | R | - | - | S | S |
| 11 | Escherichia coli | 74 | R | S | R | R | S | R | R | - | - |
| 4 | Escherichia coli | 1225 | - | S | S | - | S | S | - | - | - |
| 1 | Haemophilus influenzae | 709 | - | - | - | - | - | S | - | - | - |
| 12 | Klebsiella pneumoniae | 366 | R | S | S | S | S | S | S | - | - |
| 13 | Klebsiella pneumoniae | 13 | - | S | S | S | S | S | S | - | - |
| 13 | Klebsiella pneumoniae | 32 | - | S | S | S | S | S | S | - | - |
| 14 | Pseudomonas aeruginosa | 16 | - | S | - | - | S | S | - | - | - |
| 15 | Staphylococcus aureus | 557 | R | - | S | - | - | - | - | S | S |
| 5 | Stenotrophomonas maltophilia | 130 | R | I | R | R | R | S | S | - | - |
| 16 | Streptococcus pneumoniae | 247 | S | - | - | - | - | - | - | - | - |

*S = Sensitive, I = Intermediate, R = Resistant, - = not tested*

**Supplementary material content 3.**

*Antibiotic resistance profiles for fungal bloodstream infections*

| **Patient no.** | **Pathogen** | **Time from transplantation to BSI in days** | **Fluconazole** | **Amphotericin B** | **Anidulafungin** |
| --- | --- | --- | --- | --- | --- |
| 17 | Candida albicans | 87 | S | S | - |
| 18 | Candida albicans | 92 | S | S | S |
| 18 | Candida albicans | 132 | S | S | S |
| 19 | Candida parapsilosis complex | 925 | S | S | I |

*S = Sensitive, I = Intermediate, R = Resistant, - = not tested*
